# Toward Ameliorating Insulin Resistance: Targeting a Novel PAK1 Signaling Pathway Required for Skeletal Muscle Mitochondrial Function

**DOI:** 10.3390/antiox12091658

**Published:** 2023-08-22

**Authors:** Rekha Balakrishnan, Pablo A. Garcia, Rajakrishnan Veluthakal, Janice M. Huss, Joseph M. Hoolachan, Debbie C. Thurmond

**Affiliations:** 1 Department of Molecular and Cellular Endocrinology, Arthur Riggs Diabetes and Metabolism Research Institute, City of Hope Beckman Research Institute, 1500 E Duarte Road, Duarte, CA 91010, USA; rbalakrishnan@coh.org (R.B.); rveluthakal@coh.org (R.V.);; 2 School of Medicine, Washington University, 660 S Euclid Ave, St. Louis, MO 63110, USA; janmhuss@gmail.com

**Keywords:** type 2 diabetes, insulin resistance, skeletal muscle, mitochondria, PAK1

## Abstract

The p21-activated kinase 1 (PAK1) is required for insulin-stimulated glucose uptake in skeletal muscle cells. However, whether PAK1 regulates skeletal muscle mitochondrial function, which is a central determinant of insulin sensitivity, is unknown. Here, the effect of modulating PAK1 levels (knockdown via siRNA, overexpression via adenoviral transduction, and/or inhibition of activation via IPA3) on mitochondrial function was assessed in normal and/or insulin-resistant *rat* L6.GLUT4myc and *human* muscle (LHCN-M2) myotubes. *Human* type 2 diabetes (T2D) and non-diabetic (ND) skeletal muscle samples were also used for validation of the identified signaling elements. PAK1 depletion in myotubes decreased mitochondrial copy number, respiration, altered mitochondrial structure, downregulated PGC1α (a core regulator of mitochondrial biogenesis and oxidative metabolism) and *PGC1α* activators, p38 mitogen-activated protein kinase (p38MAPK) and activating transcription factor 2 (ATF2). PAK1 enrichment in insulin-resistant myotubes improved mitochondrial function and rescued *PGC1α* expression levels. Activated PAK1 was localized to the cytoplasm, and PAK1 enrichment concurrent with p38MAPK inhibition did not increase *PGC1α* levels. PAK1 inhibition and enrichment also modified nuclear phosphorylated-ATF2 levels. T2D *human* samples showed a deficit for *PGC1α*, and PAK1 depletion in LHCN-M2 cells led to reduced mitochondrial respiration. Overall, the results suggest that PAK1 regulates muscle mitochondrial function upstream of the p38MAPK/ATF2/*PGC1α*-axis pathway.

## 1. Introduction

Type 2 diabetes (T2D) has emerged as a clinically important and common chronic disease that is approaching epidemic proportions globally. T2D is primarily caused by the development of insulin resistance, a dysfunctional metabolic state in which the peripheral tissues (i.e., skeletal muscle, adipose and liver) are unable to effectively respond to insulin and utilize glucose from the blood. In T2D, chronic insulin resistance eventually overwhelms pancreatic insulin production and culminates in beta cell failure. This impaired metabolism can lead to numerous life-threatening health complications such as cardiovascular disease, nephropathy, neuropathy and retinopathy, contributing to increased morbidity and mortality [1,2]. Skeletal muscle is a highly metabolic tissue and the predominant site of postprandial glucose clearance, accounting for ~80% of total insulin-stimulated glucose disposal [3,4,5]. Thus, proper skeletal muscle homeostasis is essential for preserving optimal muscle mass and strength for movement and preventing metabolic diseases such as T2D and other adverse systemic effects [5,6].

Pre-type 2 diabetes is an intermediate metabolic state between normoglycemia and T2D, with impaired fasting glucose and/or impaired glucose tolerance leading to metabolic dyshomeostasis. Emerging evidence suggests that skeletal muscle insulin resistance and subsequent defective glucose/lipid utilization is the major underlying cause of metabolic dyshomeostasis, which begins in the pre-type 2 diabetes stage. Typically, within 5 years of diagnosis, individuals with prediabetes have a 50% chance of progressing to T2D [7]. Hence, improving skeletal muscle metabolic function may provide a means of restoring normoglycemia, reversing prediabetes and preventing progression to T2D.

One potential option to restore skeletal muscle insulin sensitivity is to boost mitochondrial function [8,9,10,11], given that insulin-resistant obese individuals exhibit decreased skeletal muscle mitochondrial metabolism compared to healthy lean controls [12]. Furthermore, individuals with T2D or insulin resistance are deficient for peroxisome proliferator-activated receptor γ coactivator-1α (PGC1α), the master regulator of mitochondrial biogenesis and its target genes involved in oxidative phosphorylation in their skeletal muscle [13,14,15,16]. Insulin signaling is required for mitochondrial DNA and protein synthesis, and potentially involved in regulating mitochondrial oxidative capacity and energy production [17,18]. Identifying novel skeletal muscle pathways that can restore PGC1α activity and improve mitochondrial function and oxidative capacity is necessary, so that these novel factors can be potentially harnessed therapeutically to reverse prediabetes and prevent progression to frank T2D.

We have previously reported that enrichment with the p21-activated kinase (PAK1) improves skeletal muscle insulin sensitivity in cells subjected to insulin resistance conditions [19]. PAK1 is part of an atypical signaling cascade in skeletal muscle cells, downstream of PI-3 kinase, that is linked to F-actin remodeling in non-canonical insulin signaling, which facilitates insulin-stimulated GLUT4 vesicle translocation to the cell surface [20,21]. PAK1 protein levels are associated with pre-T2D and T2D in multiple studies of T2D *human* islets and skeletal muscle, and skeletal muscle from diabetic and pre-diabetic/insulin-resistant mice, all with reduced PAK1 levels as compared with non-diabetic controls [19,22]. PAK1 deficient (*PAK1*^+/−^ and *PAK1*^−/−^) mice developed severe peripheral insulin resistance under normal chow-fed conditions [22]. Moreover, the relationship between PAK1 and glucose uptake into skeletal muscle cells has been validated [23].

PAK1 enrichment in *rat* L6.GLUT4myc myoblast/myotube cell lines was found to induce the release of muscle-derived factors, that when applied to islet beta cells, enhanced their insulin secretion capacity [19]. Although earlier studies have reported that PAK1 is indispensable for β-cell mitochondrial function/mass [6], we have yet to determine whether PAK1 plays an essential role in skeletal muscle mitochondria. Thus, this present study evaluated whether PAK1 was crucial for skeletal muscle mitochondrial function and if so, whether restoration of PAK1 in insulin-resistant skeletal muscle cells could improve its mitochondrial phenotype. We herein demonstrate that PAK1 is essential for muscle mitochondrial function, and that it is mediated through a novel p38 mitogen-activated protein kinase (p38MAPK)/activating transcription factor 2 (ATF2)/*PGC1α*-axis signaling pathway. This new signaling cascade presents putative therapeutic opportunities for reversing skeletal muscle insulin resistance in prediabetes and T2D.

## 2. Materials and Methods

### 2.1. Human Skeletal Muscle

Non-diabetic or T2D cadaveric donor skeletal muscle samples were purchased from the National Disease Research Interchange (NDRI, Philadelphia, PA, USA). The donor details are shown in Supplementary Table S1. The procurement of *human* skeletal muscle biopsies from NDRI was approved by the Institutional Review Board at the University of Pennsylvania. The participants provided informed consent prior to tissue collection. The investigation was carried out in accordance with the principles of the Declaration of Helsinki as revised in 2008. The samples were snap-frozen and kept at −80 °C until mRNA and protein extraction were carried out. The primer sequences obtained from the previous studies [19,24] were validated for sequence in the NCBI Nucleotide database. Primers were used for the detection of hPPARGC1α (forward: 5′-TACTTCAGCGAGAAGCAGGC-3′ and reverse: 5′-TCACTGCACCACTTGAGTCC-3′) and hHPRT (forward: 5′-TATGGCGACCCGCAGCCCT-3′ and reverse: 5′-CATCTCGAGCAAGACGTTCAG-3′).

### 2.2. Cell Culture

*Rat* L6.GLUT4myc skeletal muscle cells expressing c-myc-tagged GLUT4 protein were purchased from Kerafast (Boston, MA, USA, cat. # ESK202-FP) were grown as monolayers in MEM-α medium supplemented with 10% (*v*/*v*) fetal bovine serum and 1% (*v*/*v*) antibiotic–antimycotic solution (Thermo Fisher, Waltham, MA, USA). L6-GLUT4-myc myoblasts were differentiated into myotubes by incubating in MEM-α medium containing 2% fetal bovine serum and 1% antibiotic–antimycotic solution. Insulin-induced insulin resistance was performed by treating the cells with 5 nM insulin/Dulbecco’s modified Eagle’s medium (5.5 mM glucose) as previously described [25] with mild modification. For knockdown studies, small interfering RNA against PAK1 (siPAK1, Qiagen, cat. # S103082926) was transfected using RNAiMAX lipofectamine reagent (Invitrogen, Carlsbad, CA, USA). For all studies involving IPA3, L6.GLUT4myc myotubes were pre-incubated in serum-free medium containing 25 μM of IPA3 or vehicle (DMSO) for 40 min followed by insulin stimulation (100 nM for 10 or 20 min). For studies involving SB202190, L6.GLUT4myc myotubes were preincubated in serum-free medium for 40 min, followed by 10 μM SB202190 or vehicle (DMSO) for 20 min prior to insulin treatment for an additional 20 min.

For some studies, the immortalized *human* (LHCN-M2) skeletal myoblast cell line was used (a generous gift from Dr. Melissa Bowerman, Keele University, UK) [26]. The proliferating LHCN-M2 myoblasts were cultured in DMEM (Gibco)/M199 (Thermo Scientific, Waltham, MA, USA) medium (4:1) supplemented with 1% (*v*/*v*) anti-biotic/mycotic, 15% (*v*/*v*) heat inactivated FBS, 20 mM HEPES, 30 ng/mL zinc sulphate, 1.4 μg/mL vitamin B12 (Sigma-Aldrich, St. Louis, MO, USA, cat. # V6629), 55 ng/mL dexamethasone (Sigma-Aldrich, cat. # V6629), recombinant *human* hepatocyte growth factor (2.5 ng/mL) (Sigma-Aldrich, cat. # GF116), and recombinant *human* basic FGF (10 ng/mL) (BioPioneer, San Diego, CA, USA, cat. # HRP0011) on plastic culture dishes pre-coated overnight with 1% (*w*/*v*) porcine gelatin at room temperature. At around 70% confluence LHCN-M2 myoblasts were differentiated into myotubes by culturing in DMEM/M199 containing 30 ng/mL zinc sulphate, 20 mM HEPES, 1.4 μg/mL vitamin B12, and 2% (*v*/*v*) heat-inactivated horse serum; medium was refreshed every 48 h for 7–10 days until 70–80% of cells were differentiated.

Cell viability was assessed using trypan blue staining (Gibco). Cells were harvested in 1% Nonidet P-40 lysis buffer containing 25 mM HEPES (pH 7.4), 1% Nonidet P-40, 10% glycerol, 50 mM sodium fluoride, 10 mM sodium pyrophosphate, 137 mM NaCl, 1 mM sodium vanadate, 1 mM phenylmethylsulfonyl fluoride, 10 μg/mL aprotinin, 1 μg/mL pepstatin, and 5 μg/mL leupeptin and cleared of insoluble material by centrifugation at 13,000× *g* for 10 min at 4 °C. The supernatant was used for immunoblotting analyses.

### 2.3. Immunoblot Analysis

Protein lysates extracted from L6, LHCN-M2 cells and *human* tissue were resolved using 8–12% SDS-PAGE and transferred to nitrocellulose or polyvinylidene difluoride membranes (PVDF) for immunoblotting. Immunoreactive bands were detected using enhanced chemiluminescence (ECL) or ECL prime (Amersham ECL Western Blotting detection reagent, GE Healthcare, Buckinghamshire, UK) reagents and imaged using a Chemi-Doc Touch gel documentation system (Bio-Rad, Hercules, CA, USA). Quantitation of signals was performed using Image Lab v. 5.1 software and normalized to Ponceau or GAPDH levels. Antibody details are provided in Supplementary Table S2.

### 2.4. Mitochondrial Fragmentation and Superoxide Radical Detection

For analysis of superoxide radicals, L6.GLUT4myc myotubes were subject to experimental treatments as indicated in the figure legends prior to being treated with 5 μM Mitosox Red (Thermo Scientific) at 37 °C in a 5% CO_2_ incubator for 30 min. The red fluorescence that resulted from the Mitosox Red oxidation by superoxide in mitochondria was quantified using Keyence microscopy. Mitotracker green was used to detect mitochondrial fragmentation in PAK1-knockdown (KD) or -overexpressing (OE) myotubes per manufacturer instructions (Thermo Scientific).

### 2.5. Adenoviral Transduction

N-terminal GFP- or MYC-tagged hPAK1 plasmid was generated in pCDNA3 (pCDNA3-GFP/MYC hPAK1), and subcloned into the pAd5-CMV adenoviral vector. Adenoviruses were generated and purified at ViraQuest Inc. (North Liberty, IA, USA). L6.GLUT4myc myotubes were transduced at 500 MOI.

### 2.6. Mitochondrial Copy Number Evaluation

Mitochondrial DNA copy number was calculated as the ratio of mitochondrial genome to nuclear genome following real-time quantitative PCR (qPCR). DNA was isolated from L6.GLUT4myc myotubes and then analyzed by qPCR using SYBR green. The abundance of mitochondrial-encoded cytochrome B (cyt B) was compared with the nuclear 18S rRNA to produce the mitochondrial DNA abundance. The primers used were *rat* mitochondrial DNA (Cyt B forward, 5′-TCCACTTCATCCTCCCATTC-3′; reverse, 5′-CTGCGTCGGAGTTTAATCCT-3′) and nuclear DNA (18S rDNA forward, 5′-TAGAGGGACAAGTGGCGTTC-3′; reverse, 5′-CGCTGAGCCAGTCAGTGT-3′). The primer sequences obtained from the previous studies [27,28] are validated for the sequence in NCBI Nucleotide database.

### 2.7. Oxygen Consumption Rate (OCR) Measurements

L6.GLUT4myc and LHCN-M2 myotubes were grown in a 24-well Seahorse XFe24 plate (Agilent Technologies, Santa Clara, CA, USA). Cells were then transfected with siPAK1 (Qiagen, Hilden, Germany, cat. #1027418) or siCON (Qiagen, cat. #1027281) for 48 h. For reversal study, L6 myotubes were made insulin resistant with 5 nM insulin, followed by PAK1 overexpression and then tested for insulin-stimulated (100 nM, 20 min) OCR via seahorse. OCR was measured using the Seahorse XF Cell Mito Stress test kit (Agilent Technology, Santa Clara, CA, USA). Prior to the assay, the growth medium in the well of the XF cell plate was replaced with XF assay medium (Seahorse XF DMEM medium, pH 7.4, Agilent Technology) containing 1 mM sodium pyruvate, 2 mM glutamine and 5.5 mM glucose). The sensor cartridge was calibrated, and the cell plate was incubated at 37 °C for 60 min. To estimate the proportion of the basal OCR coupled to ATP synthesis using oligomycin (1 μM for L6.GLUT4myc and 0.5 μM for LHCN-M2 myotubes), an ATP synthase (Complex V) inhibitor was used. The maximal OCR that the cells could sustain was determined by use of the proton ionophore FCCP (2 μM for L6.GLUT4myc and 1 μM for LHCN-M2 myotubes). Rotenone and antimycin A (RA, 0.5 μM for L6.GLUT4myc and 0.25 μM for LHCN-M2 myotubes) were used to inhibit electron flux through CI and CIII, respectively. The cells were analyzed in the Agilent extracellular flux analysis machine (XFe24) at various intervals to assess OCR. L6.GLUT4myc and LHCN-M2 myotubes were then lysed with 1% NP-40 lysis buffer to quantify protein content for normalization of OCR.

### 2.8. RNA Isolation and qPCR

Total RNA from the L6.GLUT4myc cells were extracted using the RNeasy Plus Mini kit according to the manufacturer’s protocol (Qiagen, Hilden, Germany) and for the skeletal muscle tissue with Trizol reagent (Sigma-Aldrich, St. Louis, MO, USA). Complementary DNA was prepared using the iScript cDNA synthesis kit (Bio-Rad). The qPCR analysis was performed using the iQ™ SYBR^®^ Green Supermix. The primer sequences obtained [29] are validated for the sequence in NCBI Nucleotide database. Details of the primer sequences used are given in Supplementary Table S3.

### 2.9. Immunostaining

After appropriate treatment (transduction with PAK1 Ad-CMV myc-hPAK1 for 48 h, followed by insulin stimulation for 20 min) the cells were washed with PBS and followed by antigen unmasking using Tris-HCL buffer, pH 9.5. The antigen-retrieved cells were blocked with 1% bovine serum albumin (BSA) for an hour followed by incubation with anti-PAK1 antibody overnight at 4 °C. The sections were washed with PBS-T (phosphate-buffered saline with Tween 20) and incubated with Alexa Fluor 594-conjugated secondary antibody (Invitrogen) at room temperature for 2 h. DAPI was used to stain nuclei and the images were acquired using an LSM880 confocal microscope (Zeiss, Oberkochen, Germany). Imaris v. 10.0.1 (Bitplane, Belfast, UK) was used for 3D image reconstruction.

### 2.10. Statistics

All statistical analyses were performed using GraphPad Prism v. 9.5.1 software. The reproducibility of findings was confirmed by performing each experiment 2–5 times. One-way ANOVA/Bonferroni post hoc test or unpaired two-tailed Student’s t-test was used to compare between 2 or more groups; *p* < 0.05 was considered to be statistically significant.

## 3. Results

### 3.1. PAK1 Suppression in Skeletal Muscle Impairs Mitochondrial Structure/Function in L6.GLUT4myc Myotubes

**Figure 1:**
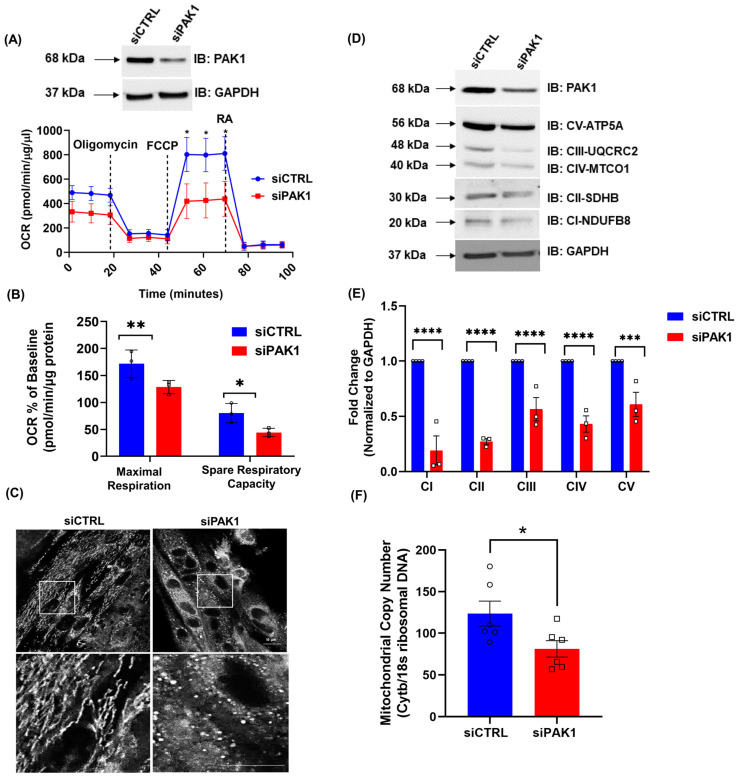
PAK1 ablation impairs mitochondrial function. (**A**–**F**) Small interfering RNA-mediated knockdown of PAK1 (siPAK1) vs. control (siCTRL) in *rat* L6.GLUT4myc myotubes (*n* = 3–6 independent experiments/group). (**A**,**B**) OCR determination with Seahorse; inset, representative Western blot of cells used for seahorse experiment. Oligomycin (blocks ATP-synthase), FCCP (dissipates the proton gradient between the matrix and inner membrane space), and antimycin A/rotenone (inhibit complexes III and I), were used in Seahorse analyses. (**C**) Confocal microscopy images of mitochondria using Mito tracker Green (pseudo colored white); scale bar = 10 μm (top), bottom inset: 9× zoom of original magnification in the boxed area. (**D**,**E**) Representative Western blot (**D**) and quantitation (**E**) of electron transport chain oxidative complex/oxphos proteins. (**F**) Quantification of mitochondrial copy number, measured by comparing the levels of cytochrome b to 18s ribosomal DNA. Circle and square symbols in the bar graphs represent the number of independent cell passages used. (**A**,**B**,**E**,**F**) Data: mean ± SEM. * *p* < 0.05, ** *p* < 0.01, *** *p* < 0.001, **** p < 0.0001 (one-way ANOVA/Bonferroni post hoc test or unpaired two-tailed Student’s *t*-test).

To test the hypothesis that PAK1 is essential for skeletal myocyte mitochondrial function, we performed siRNA-mediated silencing of the PAK1 gene in the insulin-responsive L6.GLUT4myc myoblast line, engineered to express myc-tagged GLUT4 [30]. We assessed the effect of siRNA-mediated PAK1 knockdown on mitochondrial energetics in differentiated L6.GLUT4myc using the seahorse flux analyzer to measure OCR. We found a significant decrease of maximal respiration and spare respiratory capacity in PAK1-silenced myotubes, compared to siRNA scrambled control transfected cells (siCTRL) (Figure 1A,B). In addition, fragmented and punctate mitochondria were predominantly found in siPAK1 cells, whereas control cells displayed healthy interconnected, elongated mitochondrial networks (Figure 1C). There was a concomitant significant decrease in proteins involved in oxidative phosphorylation in PAK1 knockdown cells (Figure 1D,E), suggesting that PAK1 deficiency may impact mitochondrial activity. Furthermore, we found that myotubes transfected with siPAK1 also had significantly reduced mitochondrial DNA copy number (Figure 1F). Gene expression analysis showed siPAK1-treated myotubes expressing significantly reduced *COX4* mRNA levels, but no significant changes were observed in the other major mitochondrial regulatory genes compared with siCTRL-treated myotubes (Supplementary Figure S1A). These data demonstrate that PAK1 is indispensable for skeletal muscle mitochondrial capacity and function.

### 3.2. PAK1 Enrichment in Insulin-Resistant L6 Myotubes Protects from Mitochondrial Damage

**Figure 2:**
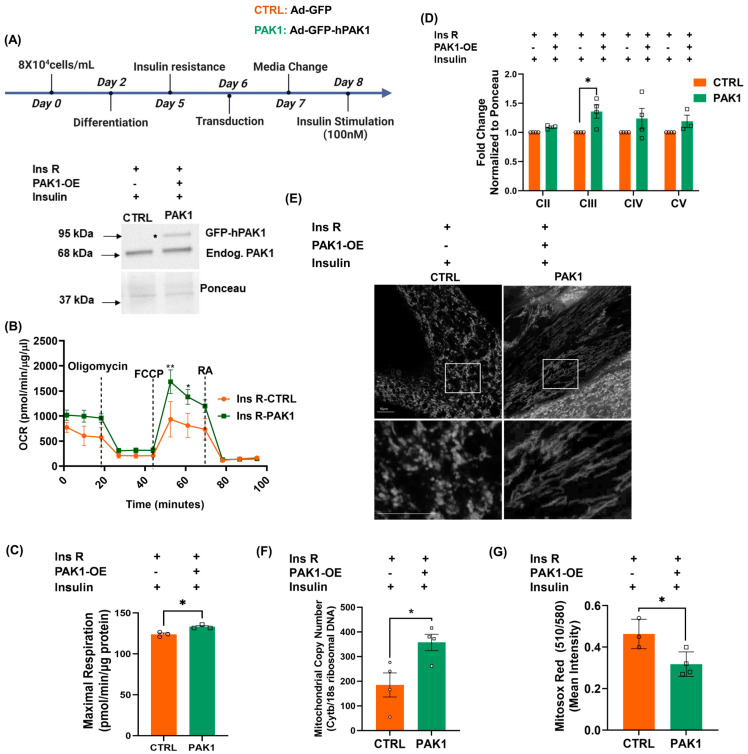
PAK1 enrichment restores mitochondrial structure/function. (**A**) Schematic of insulin resistance (Ins R) reversal approach using *rat* L6.GLUT4myc myotubes. Post-Ins R L6.GLUT4myc myotubes were transduced with Ad-GFP-hPAK1 (PAK1 overexpression) or CTRL-Ad-GFP (control). Transduced-Ins R cells were stimulated with insulin for 20 min and were used in (**B**–**G**). *n* = 3–4 independent cell passages per group. (**B**–**G**) PAK1-OE, PAK1 overexpression. (**B**) Seahorse analysis of OCR; inset, representative Western blot of cells used for seahorse. (**C**) OCR quantitation (% of baseline). (**D**) Western blot quantitation of electron transport chain oxidative complex/oxphos proteins. (**E**) Confocal microscopy images of mitochondria using Mito tracker Green (pseudo colored white); scale bar = 10 μm (top), bottom inset: 9× zoom of original magnification in the boxed area. (**F**) Quantification of mitochondrial copy number by comparing the levels of cytochrome b to 18s ribosomal DNA. (**G**) Quantification of superoxide radical levels. Circle and square symbols in the bar graphs represent the number of independent cell passages used. (**B**–**D**,**F**,**G**) Data: mean ± SEM. * *p* < 0.05, ** *p* < 0.01 (one-way ANOVA/Bonferroni post hoc test or unpaired two-tailed Student’s *t*-test).

Given that PAK1 is essential for skeletal muscle mitochondrial function, we next examined whether PAK1 enrichment in myotubes pre-cultured under conditions to mimic the insulin-resistant (Ins R) disease state had the capacity to reverse mitochondrial impairments. For reversal studies, we induced Ins R in L6.GLUT4myc myotubes via chronic exposure to 5 nM insulin prior to GFP-tagged *human* PAK1 overexpression or GFP-tagged control-vector expression (Figure 2A) and the Ins R paradigm was validated via assessing insulin-stimulated Akt phosphorylation (Supplementary Figure S2A). Interestingly, PAK1 enrichment (PAK1 OE) in Ins R myotubes improved mitochondrial OCR (Figure 2B) and significantly increased maximal respiration (Figure 2C) compared to control. Consistent with improved mitochondrial function, mitochondrial complex III protein (CIII) was also significantly increased in PAK1-enriched Ins R myotubes (Figure 2D, Supplementary Figure S2B). Interestingly, structural analyses of MitoTracker Green-stained PAK1-enriched myotubes by confocal microscopy revealed healthy interconnected and elongated mitochondrial networks, whereas control myotubes showed fragmented mitochondrial structure (Figure 2E). The mitochondrial copy number was increased in PAK1-enriched Ins R myotubes (Figure 2F). Moreover, PAK1-enriched Ins R myotubes exhibited decreased superoxide radical production, as detected using MitoSox Red (Figure 2G, Supplementary Figure S2B). Together, these data indicate that PAK1 enrichment in insulin-resistant myotubes restores mitochondrial structure and function in Ins R skeletal myocytes.

### 3.3. PAK1 Regulates PGC1α Gene Expression

**Figure 3:**
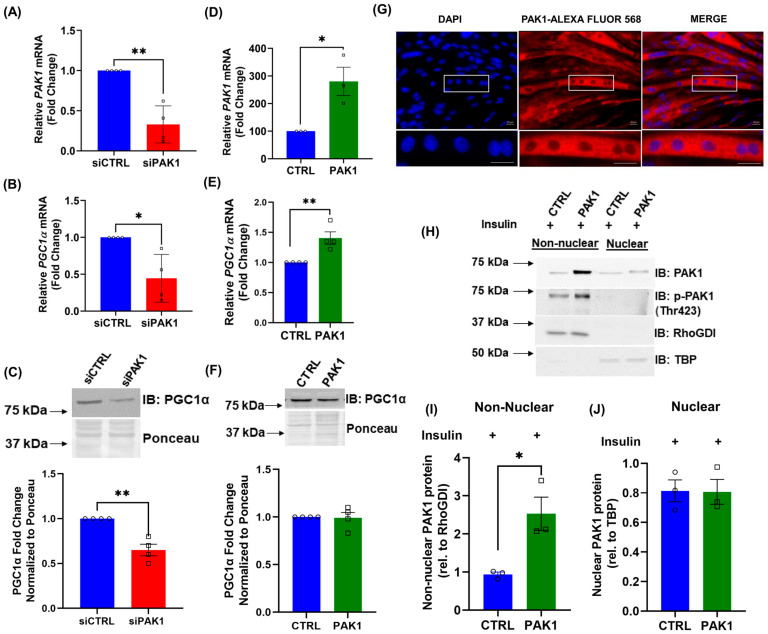
PAK1 facilitates *PGC1α* gene expression. (**A**–**C**) PAK1-depleted (siPAK1) or control-transfected (siCTRL) L6.GLUT4myc myotubes showing qPCR quantification of *PAK1* mRNA expression (**A**) and corresponding *PGC1α* mRNA expression (**B**). Representative immunoblot (top) and quantitation (bottom) of PGC1α protein levels (**C**). (**D**–**F**) GFP-tagged PAK1-enriched (PAK1) or control (CTRL) L6.GLUT4myc myotubes showing qPCR quantification of *PAK1* mRNA expression (**D**) and corresponding *PGC1α* mRNA expression (**E**). Representative immunoblot (top) and quantitation (bottom) of PGC1α protein levels (**F**). (**G**–**J**) L6.GLUT4myc myotubes transduced to express PAK1 with Ad-MYC-hPAK1 (PAK1-OE) post-insulin stimulation for 20 min. CTRL, control vector. (**G**) Confocal immunofluorescent images showing PAK1 localization; scale bar = 20 μm (top), bottom inset: 6× zoom of original magnification in the boxed area. Blue: DAPI nuclear stain. Red: PAK1. (**H**–**J**) Representative immunoblot (**H**) and quantitation of PAK1 localized to the non-nuclear (**I**) vs. nuclear (**J**) fraction. In each independent experiment, control was set equal to 100% and other samples normalized thereto. Bars represent the mean ± SEM from 3–4 independent experiments. Circle and square symbols in the bar graphs represent the number of independent cell passages used. * *p* < 0.05, ** *p* < 0.01 (unpaired two-tailed Student’s *t*-test). *β-Actin* was used as the loading control for normalization of gene expression analysis and ponceau was used as the loading control for normalization of immunoblots.

To determine how PAK1 enrichment had the ability to ameliorate mitochondrial dysfunction and structural damage mediated by insulin resistance in skeletal myotubes, we evaluated the mRNA and protein levels of PGC1α in these myotubes. PGC1α is the master regulator of skeletal muscle mitochondrial function [31,32]. Since *PGC1α* and oxidative phosphorylation genes are reduced in *human* T2D and glucose intolerant skeletal muscle [13,14], and PAK1 is implicated in whole body glucose homeostasis in vivo [22,33], we evaluated whether alterations to PAK1 levels might impact PGC1α levels. PAK1-depleted (siPAK1) myotubes displayed significantly reduced PGC1α mRNA and protein levels (Figure 3A–C). While PGC1α protein levels were unchanged (as expected due to PGC-1α protein having a short half-life and being subject to rapid changes), PAK1-enriched myotubes correlated with upregulated *PGC1α* gene expression (Figure 3D–F).

To further understand the linkage between PAK1 signaling and *PGC1α* gene expression, we investigated the potential for PAK1 localization to the nuclear compartment. PAK1 can translocate to the nuclear compartment of several cell types in response to activating stimuli [34]. Nevertheless, in myotubes, PAK1 was found principally to be localized to the non-nuclear compartments by confocal microscopy (Figure 3G), and PAK1 overexpression did not induce significant changes to nuclear localized PAK1 protein levels after 20 min of insulin stimulation (Supplementary Movies S1 and S2). We also explored a biochemical approach by fractionating PAK1 (myc-tagged PAK1, 70 kDa)-enriched myotubes into nuclear versus non-nuclear compartments (Figure 3H–J). In contrast to control-transduced myotubes, PAK1 protein levels were increased in non-nuclear compartments with PAK1 overexpression (Figure 3H,I). Control-vector and PAK1 overexpression under insulin stimulation conditions did not show any changes in nuclear PAK1 levels (Figure 3H,J) or activated/phosphorylated PAK1 levels (Figure 3H) in the nuclear compartment. This suggests that insulin-induced PAK1 activation does not directly induce nuclear-localized gene expression changes in this cell type.

### 3.4. PAK1 Regulates p38MAPK/ATF2/PGC1α-Axis Signaling

**Figure 4:**
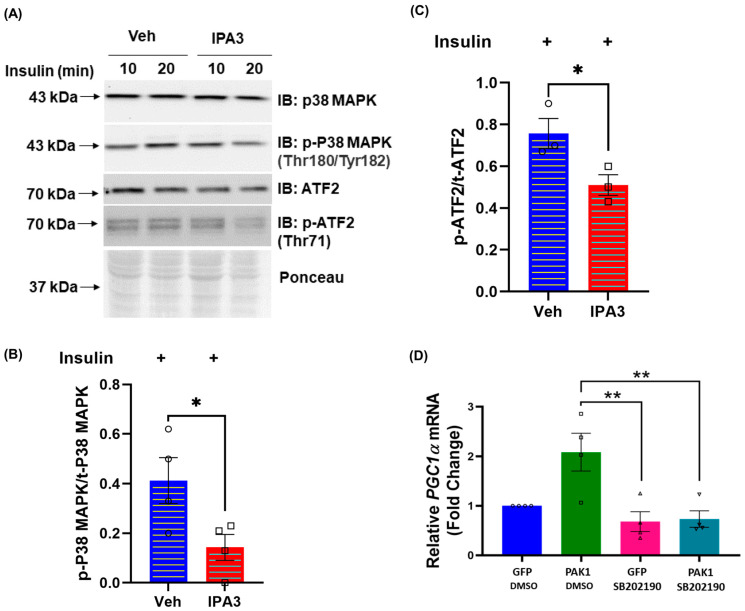
PAK1 regulates p38MAPK/ATF2/*PGC1α*-axis signaling. (**A**–**C**) L6.GLUT4myc myotubes were pretreated with vehicle (DMSO, veh) or 25 μM IPA3 for 40 min and stimulated with 100 nM insulin for an additional 10 or 20 min. (**A**) Whole-cell lysates of L6.GLUT4myc myotubes were analyzed for stated proteins. Phosphorylated, p. Graphs show quantification of phosphorylated/total protein ratio (**B**,**C**). (**D**) Quantification of *PGC1α* gene expression ± PAK1 enrichment (using Ad-GFP-hPAK1) and ±p38MAPK inhibitor (SB202190) treatment in myotubes. For each independent experiment, control was set equal to 100% and other samples normalized thereto. *β-Actin* was used as the loading control for normalization of gene expression analysis. GFP, empty vector. DMSO, control treatment. Symbols in the bar graphs represent the number of independent cell passages used. Bars represent the mean ± SEM from 3–4 independent experiments. * *p* < 0.05, ** *p* < 0.01 (one-way ANOVA/Bonferroni post hoc test or unpaired two-tailed Student’s *t*-test was used for statistical analysis).

The lack of PAK1 nuclear localization and absence of insulin-stimulated PAK1 in the nucleus suggested that insulin-stimulated PAK1 activation occurred outside the nucleus and promoted the observed upregulation of *PGC1α* expression. To test this, we used the pharmacological inhibitor of the group I PAK proteins (PAK1, PAK2, and PAK3; PAK3 is absent in skeletal muscle) [35,36]. IPA3 is a small molecule allosteric inhibitor of PAK activation. We and others previously reported a decrease of insulin-stimulated PAK1 phosphorylation with IPA3 treatment [23,37]. Indeed, IPA3-mediated inhibition of PAK1 activation acutely decreased the activation of proteins known to transcriptionally regulate PGC1α, namely p38MAPK and its downstream effector protein, ATF2 (Figure 4A–C). PAK1 overexpression in p38MAPK inhibitor-treated cells (SB202190) could not rescue *PGC1α* to normal levels, further suggesting that PAK1-induced *PGC1α* gene expression is mediated via p38MAPK (Figure 4D).

### 3.5. PAK1 Regulates the Nuclear Translocation of ATF2

**Figure 5:**
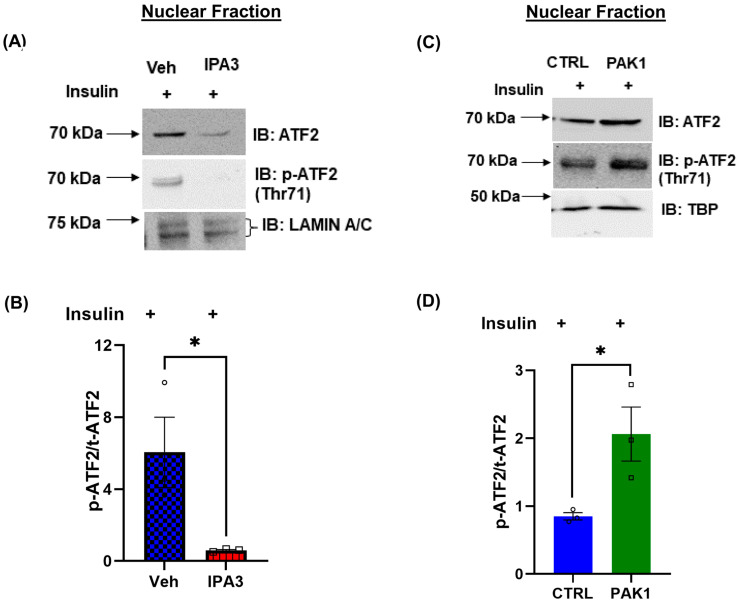
PAK1 regulates the nuclear translocation of ATF2. (**A**,**B**) Representative immunoblot (**A**) and quantification (**B**) of phosphorylated (p)-ATF2 vs. total (t) ATF2 in nuclear extracts of IPA3-treated *rat* L6.GLUT4myc myotubes and stimulated with insulin for 20 min. (**C**,**D**) Representative immunoblot (**C**) and quantification (**D**) of p-ATF2 vs. t-ATF2 in nuclear fraction of PAK1-enriched (using Ad-MYC-hPAK1) L6.GLUT4myc myotubes stimulated with insulin for 20 min. (**B**,**D**) Data: mean ± SEM. (**A**–**D**) *n* = 3 independent cell passages per group, circle and square symbols in the bar graphs represent the number of independent cell passages used. * *p* < 0.05 (unpaired two-tailed Student’s *t*-test).

Skeletal muscle p38MAPK activation during contractile activity in mice, or by overexpression of p38MAPK in C2C12 myocytes, triggers nuclear localization of the transcription factor ATF2 [38]. To determine whether PAK1 activation is required for ATF2 nuclear localization, myotubes were acutely treated with IPA3 or vehicle control, and myotubes were fractionated for quantitation of total ATF2 and phosphorylated-ATF2 (p-ATF2) in the nuclear compartment. ATF2 levels were reduced in nuclear fractions of IPA3-treated myotubes, relative to vehicle-treated myotubes, and pATF2 was largely undetectable in IPA3-treated nuclear fractions (Figure 5A,B). By contrast, PAK1 enrichment yielded increased ATF2 localization to the nuclear compartment, as well as increased p-ATF2, compared to control myotubes (Figure 5C,D). These data suggest that PAK1 activates *PGC1α* gene expression through ATF2 nuclear translocation and activation.

### 3.6. T2D Human Muscle and PAK1-Deficient Myotubes Exhibit Impaired Mitochondrial Function and PGC1α Expression

**Figure 6:**
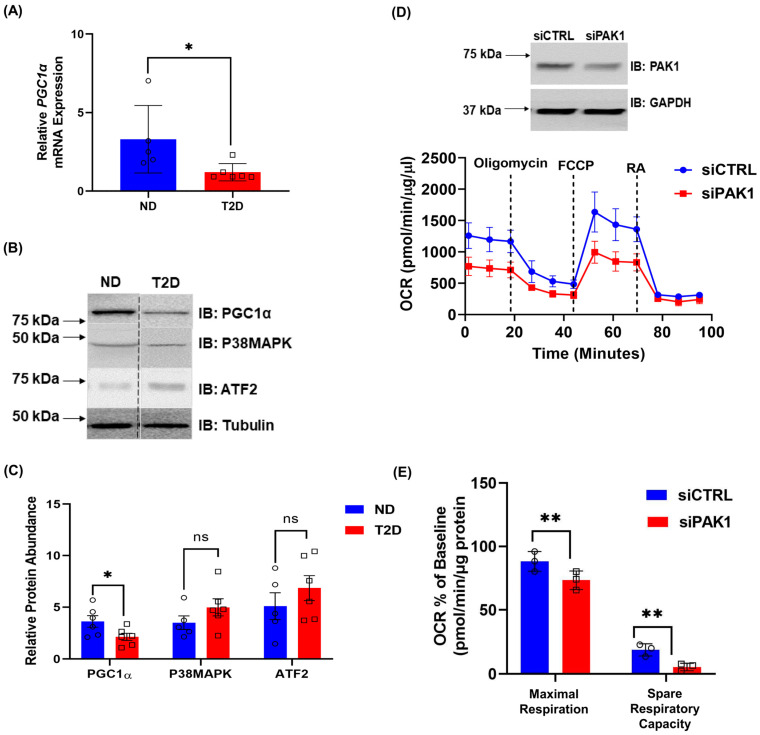
p38MAPK, ATF2, and PGC1α levels are decreased in *human*s with T2D. (**A**) Quantification of *PGC1α* mRNA in skeletal muscle samples from individuals with T2D (*n* = 6) compared to non-diabetic (ND, *n* = 5) individuals. (**B**,**C**) Representative immunoblot (**B**) and quantification (**C**) of p38MAPK, ATF2 and PGC1α in *human* T2D (*n* = 6) and ND (*n* = 5) skeletal muscle samples. Vertical dashed lines indicate the splicing of lanes from within the same gel exposure. Two-tailed unpaired Student’s *t*-test, * *p* < 0.05. (**D**,**E**) *Human* LHCN-M2 myotubes ± PAK1 knockdown. siCTRL, control siRNA. siPAK1, PAK1 siRNA. *n* = 3 independent cell passages per group, circle and square symbols in the bar graphs represent the number of independent cell passages used. (**D**) Seahorse analysis of OCR (bottom) and representative immunoblot of cells in seahorse (top). (**E**) Quantification of OCR % of baseline. ** *p* < 0.01, ns., not significant (one-way ANOVA/Bonferroni post hoc test). (**A**,**C**–**E**) Data: mean ± SEM.

PAK1 protein is significantly decreased in *human* T2D skeletal muscle compared to the non-diabetic controls [19]. To determine if PAK1 loss is correlated with any losses in the signaling components identified in our *rat* L6.GLUT4myc myotube studies, activation and protein levels of p38MAPK, ATF2, and PGC1α were evaluated using the same *human* non-diabetic and T2D skeletal muscle samples. Indeed, decreased levels of *PGC1α* mRNA and protein were found in the PAK1-deficient T2D *human* muscle samples (Figure 6A–C). However, total protein levels of p38MAPK, ATF2 and oxphos complex proteins showed no significant changes in T2D compared to non-diabetic muscle samples (Figure 6B,C, Supplementary Figure S3A,B). Of note, the *human* donor muscle was collected under random conditions (i.e., donor cadaveric tissues were not from individuals in a defined fasted or fed state, nor insulin-stimulated per se), and hence phosphorylated levels of p38MAPK or ATF2 were not quantified. Correlation analyses of abundances of PGC-1α with age, BMI, PAK1 protein (Supplementary Figure S3C–E), or of oxphos complex protein abundances with age or BMI (Supplementary Figure S3F,G) showed no significant associations. Similarly, correlation analyses of abundances of PAK1, P38MAPK, or ATF2 proteins with age or BMI (Supplementary Figures S4–S6), or between PAK1 protein abundance with P38MAPK or ATF2 protein abundances (Supplementary Figure S7) showed no significant associations. We further examined the impact of siPAK1 in the *human* LHCN-M2 skeletal myotube line [39]. Consistent with our *rat* myotube results, siPAK1-treated *human* myotubes exhibited significantly impaired OCR (Figure 6D,E), indicating the importance and relevance of PAK1 for mitochondrial function in *human* skeletal muscle.

## 4. Discussion

This study revealed a novel role for PAK1 in skeletal muscle mitochondrial biogenesis, metabolism and structure. Intriguingly, PAK1 depletion in skeletal muscle myotubes was associated with decreased mitochondrial copy number, OCR, reduced levels of proteins involved in oxidative phosphorylation and fragmented mitochondrial structure. In contrast, PAK1 enrichment in insulin-resistant *rat* L6 myotubes showed improved mitochondrial copy number, OCR and increased oxidative phosphorylation-associated protein levels. Further, our findings demonstrate that PAK1 regulates the p38MAPK/ATF2/*PGC1α*-axis signaling pathway suggesting that PAK1 contributes to muscle mitochondrial metabolism through *PGC1α* gene activation. In this study, we report the existence of new signaling elements downstream of PAK1 that regulate *PGC1α* gene expression, the known key regulator of skeletal muscle mitochondrial function. Moreover, the in vitro models (*rat* cell line: L6.GLUT4myc, and *human* cell line: LHCN-M2) used to study muscle physiology under insulin-resistant conditions have provided invaluable insights into the intracellular mechanisms operating in prediabetes. Of note, we also validated the trend showing decreased PAK1 and PGC1α in T2D *human* skeletal muscle samples and demonstrated reduced mitochondrial activity in PAK1-deficient LHCN-M2 *human* myotubes, providing applicability of our findings to *human*s.

A key finding in this current study is that PAK1 is required for skeletal muscle mitochondrial function and structure. Indeed, a recently emerging role for PAK1 is in the mitochondria of other cells. For example, it was found that PAK1-deficient β-cells had decreased mitochondrial copy number, mitochondrial respiration, disrupted redox balance, elevated ROS, diminished levels of the mitochondrial electron transport chain complex proteins and increased apoptosis [6]. In addition, PAK inhibition by IPA3 in leukemia cell lines significantly lowered OCR, revealing roles for PAK1 and PAK2 in the metabolic regulation of both mitochondrial respiration and aerobic glycolysis [40]. Moreover, a recent report indicated that PAK2 stimulates the activity of the cancer-specific isoform of pyruvate kinase which directs the cell metabolism to aerobic glycolysis [41]. The present study validated that the ablation of skeletal muscle PAK1 resulted in mitochondrial anomalies. Indeed, mitochondria are localized at I-bands in skeletal muscle [42], and disrupted mitochondrial distribution can cause cell death [43,44,45]. In support of this notion, skeletal muscle of PAK1 KO mice crossed with PAK2 myoD1 conditional knockout mice harbor reduced mitochondrial energetics and megaconial mitochondria that lack central mitochondrial activity [46].

Our current findings established that PAK1 enrichment in skeletal muscle had the capacity to reverse mitochondrial defects in insulin-resistant myotubes, via a mechanism involving activation of p38MAPK, followed by ATF2 activation, to increase *PGC1α* expression. *Human* T2D and insulin resistance are associated with impaired skeletal muscle mitochondrial function [17,47,48], decreased expression of oxidative metabolism genes and proteins [13,14,49], reduced mitochondrial size and copy number [8], and reduced levels of PAK1 [22]. Our current findings, using T2D *human* skeletal muscle and PAK1-depleted *human* and *rat* myotube cell lines, expand the current knowledge by demonstrating a requirement for PAK1 signaling in driving *PGC1α* expression. This observation is highly relevant given that PGC1α is a core regulator of skeletal muscle mitochondrial biogenesis and oxidative metabolism [32,50,51]. Interestingly, PAK1 deficiency in *human* T2D muscle or induced using siRNA knockdown in L6 myotubes, reduced expression of PGC1α gene and protein. Conversely, adenovirus-mediated PAK1 restoration in insulin-resistant L6 myotubes increased *PGC1α* gene expression only—protein levels were unchanged. This finding is consistent with a recent study in healthy adult muscle, wherein insulin stimulation increased muscle *PGC1α* mRNA without significantly altering PGC1α protein levels [10,52]. Further studies are required to delineate the mechanisms involved.

We determined in the current study that the signaling triggered by PAK1 to *PGC1α* was not via a nuclear PAK1 presence. Although PAK1 has been shown in other cell types to translocate to the nucleus [53,54,55], PAK1 overexpression in *rat* L6.GLUT4myc myotubes caused no significant increase in the level of nuclear-localized PAK1 protein. This suggests that PAK1-induced increase in PGC1α is not due to increased PAK1 transactivation of *PGC1α* gene expression. Instead, our PAK1 knockdown and IPA3 inhibitor studies provide evidence for PGC1α regulation in myotubes by PAK1 via modulation of PGC1α transcriptional regulators—namely, p38MAPK and ATF2. Consistent with this, inhibition of p38MAPK activity using SB202190 reduced *PGC1α* gene levels and overexpression of PAK1 in SB202190-treated cells did not rescue *PGC1α* mRNA levels, suggesting that PAK1 modifies *PGC1α* expression via p38MAPK signaling. In addition, IPA3 decreased phosphorylated ATF2 levels and PAK1 overexpression increased phosphorylated ATF2 levels in the nuclear fraction, the locale where ATF2 is known to promote *PGC1α* gene expression [56]. Prior findings have implicated activated MAPK-mediated changes in muscle gene expression in response to exercise, and members of the MAPK pathways are potential PAK1 targets [57,58,59], further supporting our model. It was also reported that passive stretch increases phosphorylation of skeletal muscle PAK1/2 activation of p38MAPK in mice [60]. Overall, our new findings reveal that a reduction in PAK1 abundance might promote increased mitochondrial anomalies and increase susceptibility to prediabetes and subsequent progression to T2D. Therefore, enhancing skeletal muscle-specific PAK1 signaling could ameliorate muscle metabolic dysfunction, improve insulin sensitivity, reverse prediabetes and prevent progression to T2D.

## 5. Conclusions

We conclude that PAK1 is essential for muscle mitochondrial function, and it is mediated through a p38MAPK/ATF2/*PGC1α*-axis signaling pathway, providing a new signaling cascade for potential therapeutic interventional strategies to skeletal muscle insulin resistance in prediabetes and T2D.

## Data Availability

Data is contained within the article.

## Supplementary Materials

The following supporting information can be downloaded at: https://www.mdpi.com/article/10.3390/antiox12091658/s1, Figure S1: Skeletal Muscle Mitochondrial Regulatory Genes in PAK1 Knockdown vs. Overexpressing L6 Myotubes; Figure S2: Changes in Mitochondrial Complex Protein and Reactive Oxygen Species (ROS) levels with PAK1 Enrichment; Figure S3: Mitochondrial Oxphos complex and PGC1α Abundance in *Human* Skeletal Muscle Samples; Figure S4: Evaluation of PAK1 Abundance Correlation in *Human* Skeletal Muscle Samples; Figure S5: Evaluation of P38MAPK Abundance Correlation in *Human* Skeletal Muscle Samples; Figure S6: Evaluation of ATF2 Abundance Correlation in *Human* Skeletal Muscle Samples; Figure S7: Evaluation of ATF2 Abundance Correlation in *Human* Skeletal Muscle Samples; Table S1: Non-diabetic and T2D human skeletal muscle donor information; Table S2: Details of the antibodies used; Table S3: Primer Details; Movie S1: PAK1 enrichment and insulin stimulation did not alter nuclear-localized PAK1 levels. L6-GLUT4myc myotubes transduced with Ad-CMVGFP (Control) were insulin (100 nM, 20 min) stimulated after 48 h of transduction; Movie S2: PAK1 enrichment and insulin stimulation did not alter nuclear-localized PAK1 levels. L6-GLUT4myc myotubes transduced with Ad-CMVmyc-hPAK1 (PAK1) were insulin (100 nM, 20 min) stimulated after 48 h of transduction.
