# Supplementary material for: Toward Ameliorating Insulin Resistance: Targeting a Novel PAK1 Signaling Pathway Required for Skeletal Muscle Mitochondrial Function
Source: Antioxidants (Basel). 2023 Aug 22;12(9):1658. doi: 10.3390/antiox12091658 (PMC10525748; doi:10.3390/antiox12091658)
Supplement: Supplementary file 1 [file antioxidants-12-01658-s001.zip › antioxidants-2510412-supplementary.docx]

**Supplemental Information**

Toward Ameliorating Insulin Resistance: Targeting a Novel PAK1 Signaling Pathway Required for Skeletal Muscle
Mitochondrial Function

Rekha Balakrishnan ^1^, Pablo A. Garcia ^1^, Rajakrishnan Veluthakal ^1^, Janice M. Huss ^2^, Joseph M. Hoolachan ^1^
and Debbie C. Thurmond ^1,^*

^1^ Department of Molecular and Cellular Endocrinology, Arthur Riggs Diabetes and Metabolism Research Institute, City of Hope Beckman Research Institute, 1500 E Duarte Road, Duarte, CA 91010, USA;
rbalakrishnan@coh.org (R.B.); pgarcia@coh.org (P.A.G.); rveluthakal@coh.org (R.V.);
jhoolachan@coh.org (J.M.H.)

^2^ School of Medicine, Washington University, 660 S Euclid Ave, St. Louis, MO 63110, USA; janmhuss@gmail.com

***** Correspondence: dthurmond@coh.org

**Table of Contents:**

**Supplementary Table S1** .**S2**

**Supplementary Table S2** .**S3**

**Supplementary Table S3** .**S4**

**Supplementary Figure S1 S5**

**Supplementary Figure S2** **S6**

**Supplementary Figure S3** **S7**

**Supplementary Figure S4** **S8**

**Supplementary Figure S5 S9**

**Supplementary Figure S6** **S10**

**Supplementary Figure S7 S11**

**Supplementary Movies S1, S2 S12**

| **Sample** | **NDRI#** | **Age** | **Race** | **Gender** | **BMI** | **Condition** |
| --- | --- | --- | --- | --- | --- | --- |
| 1 | ND09743 | 46 | Caucasian | M | 34.9 | ND |
| 2 | ND09744 | 62 | Caucasian | M | 28.2 | ND |
| 3 | ND13347 | 78 | Caucasian | M | 26.4 | ND |
| 4 | ND09749 | 49 | Caucasian | F | 21.6 | ND |
| 5 | ND13230 | 68 | Caucasian | F | 40.2 | ND |
| 6 | ND13216 | 54 | Caucasian | M | 31.3 | ND |
| 7 | ND09703 | 68 | Caucasian | F | 29.9 | T2D |
| 8 | ND09705 | 65 | Caucasian | M | 33.8 | T2D |
| 9 | ND09706 | 45 | Caucasian | M | 44.5 | T2D |
| 10 | ND09754 | 67 | Caucasian | M | 32.3 | T2D |
| 11 | ND13363 | 59 | Caucasian | M | 41.5 | T2D |
| 12 | ND13239 | 58 | Caucasian | M | 50.3 | T2D |

ND= Non-diabetic individuals; T2D= Type 2 diabetic individuals; BMI= Body mass index; M= Male, F= Female; NDRI# = National disease research interchange (NDRI) sample number, provided muscle source as quadriceps or ‘leg muscle’.

| **Antibody** | **Vendor** | **Catalog Number** |
| --- | --- | --- |
| PAK1 | Cell Signaling | 2602S |
| p-PAK1 | Cell Signaling | 2601 |
| P38 MAPK | Cell Signaling | 9212S |
| p-P38 MAPK | Cell Signaling | 9211S |
| AKT | Cell Signaling | 2920S |
| p-AKT | Cell Signaling | 4060S |
| TBP | Cell Signaling | 44059S |
| ATF2 | Cell Signaling | 35031S |
| p-ATF2 | Cell Signaling | 24329S |
| GAPDH | Invitrogen | AM4300 |
| Oxphos cocktail | Abcam | ab110413 |
| PGC1α | Novus Biologicals | NBP3-08971 |

| **Gene** | **Forward (5’→3’)** | **Reverse (5’→3’)** |
| --- | --- | --- |
| SDHA | GCCACTCACTCTTACACACC | GCACTCCCCATTTTCCATC |
| UCP3 | GTCAAGCAGTTCTACACCCC | TTTCCTCTCGCCTCCAGTTC |
| ANT1 | TCATCTACAGAGCTGCCTAC | TCATCATCCTACGACGGAC |
| ND4 | GAGGCAACCAAACAGAACGC | ATCATGTTGAGGGTAGGGGGT |
| COX4 | AATGTTGGCTACCAGGGCAC | GGGTAGTCACGCCGATCAAC |
| β-ACTIN | TGGAGAAGATTTGGCACCACAC | CAGAGGCATACAGGGACAACAC |
| PPARGC1A | TGAACTACGGGATGGCAAC | AAGAGCAAGAAGGCGACAC |
| eGFP-hPAK1 | CATGGTCCTGCTGGAGTTC | TCTTGAATGTCTAGGCCGTTATT |

**F)**

**G)**

**H)**

**I)**

**A)**


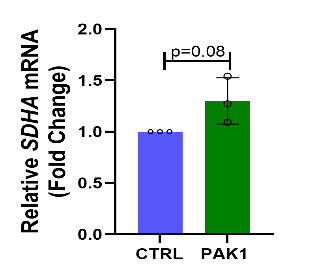

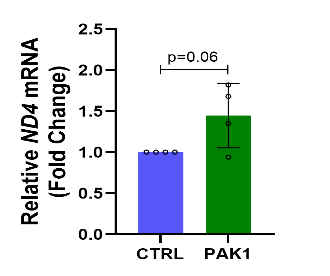

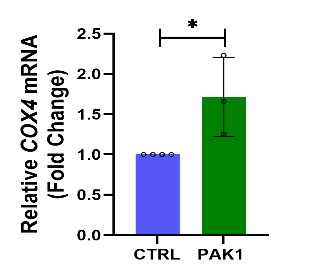

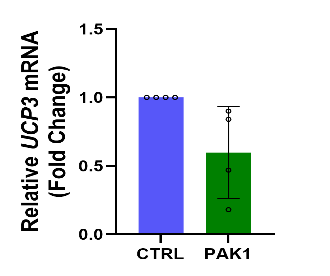

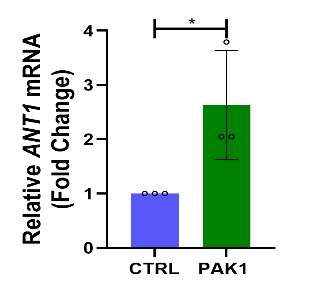

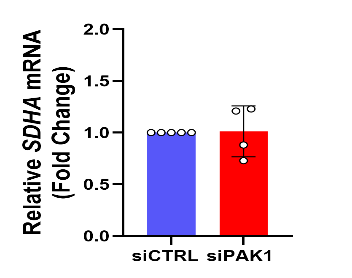

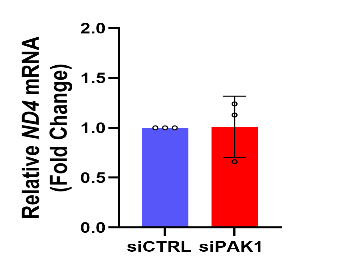

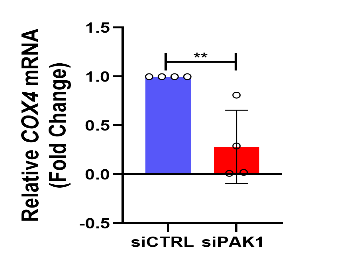

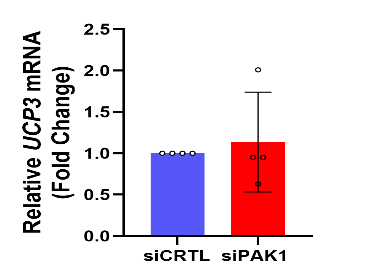

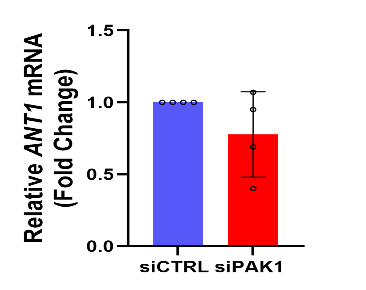


**B)**

**C)**

**D)**

**J)**

**E)**

**Supplementary Figure S1. Skeletal Muscle Mitochondrial Regulatory Genes in PAK1 knockdown vs. overexpressing L6 Myotubes**. (A-E) PAK1 knockdown with small interfering RNA (siPAK1). siCTRL, Control. (F-J) PAK1 overexpression with adenovirus. CTRL, control. (A-J) Data: mean ± SEM. N= 3-4 independent cell passages per group. **p* < 0.05, ***p* < 0.01 (unpaired two-sided Student t test).

**
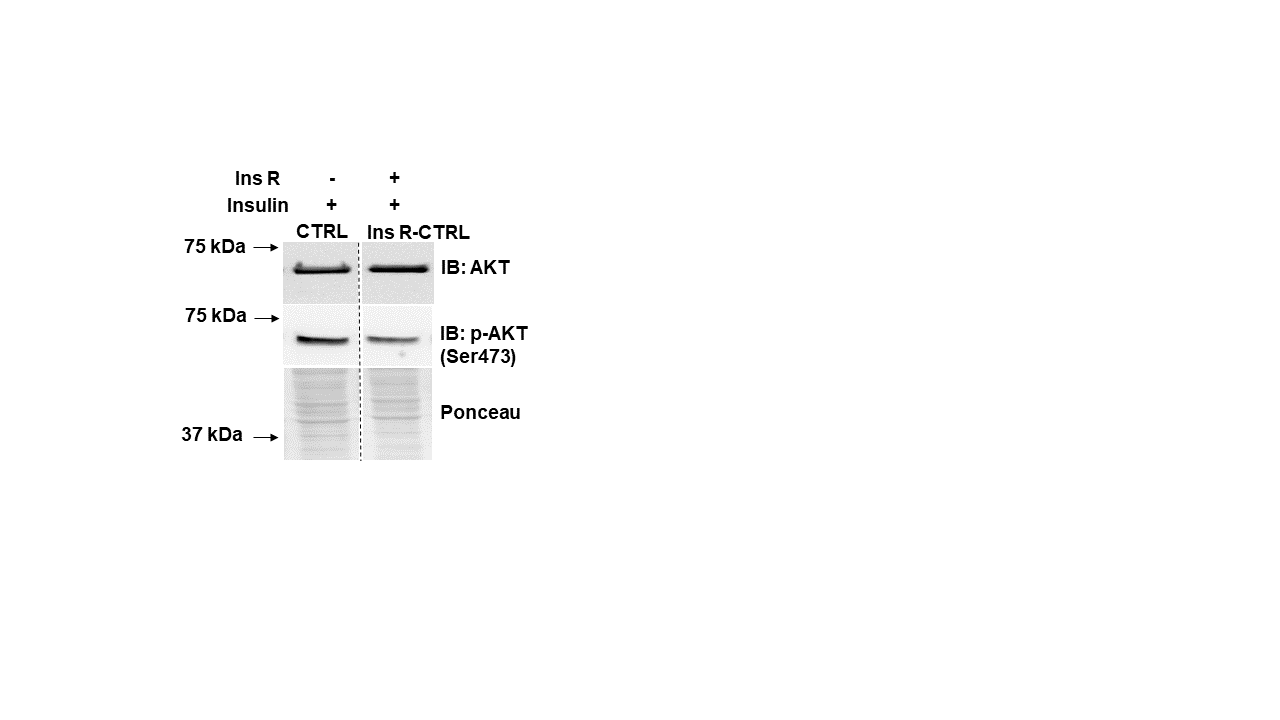
**

**A)**

**B)**

**
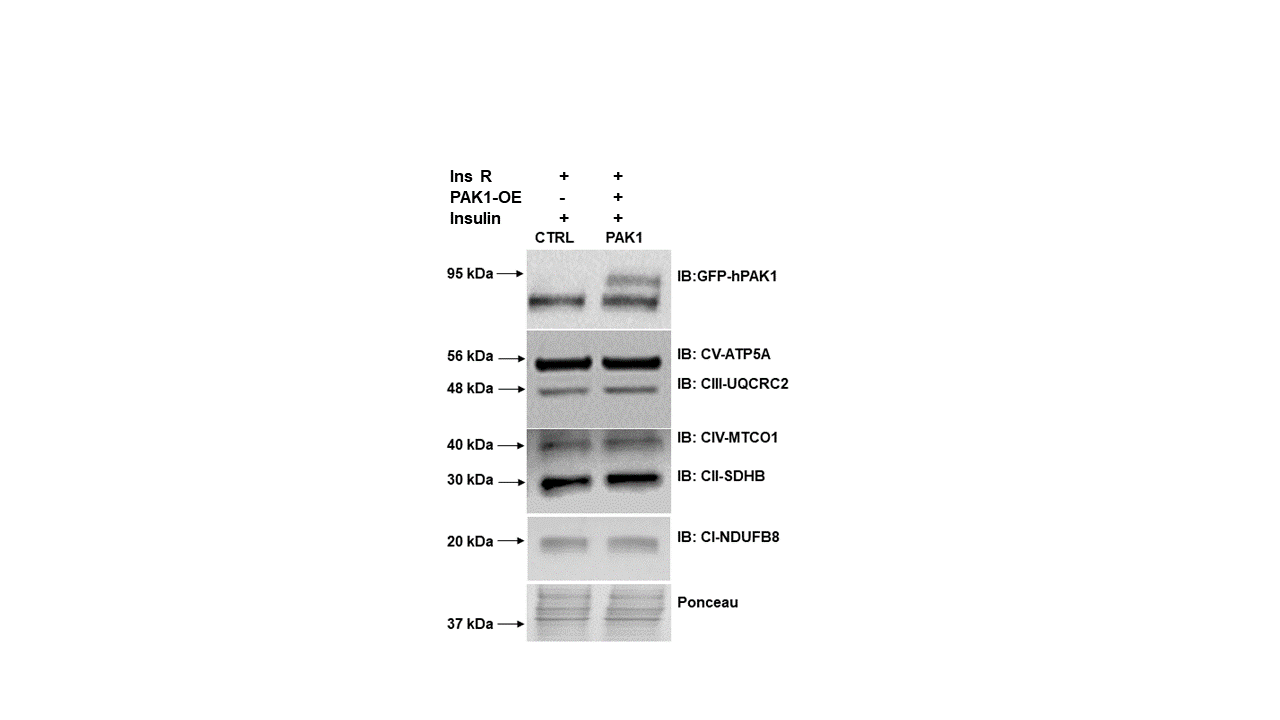
**


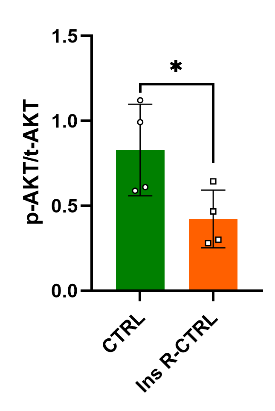


**C)**

**DAPI MITOSOX RED DAPI + MITOSOX RED**


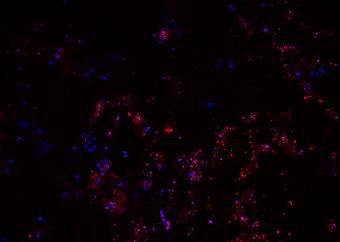

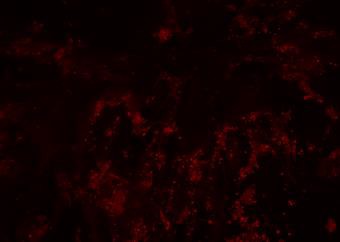

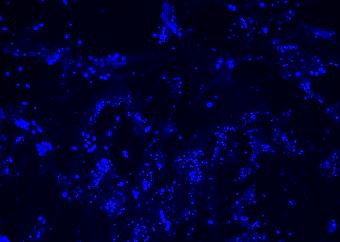

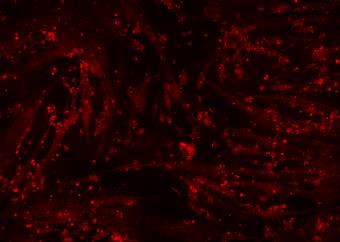

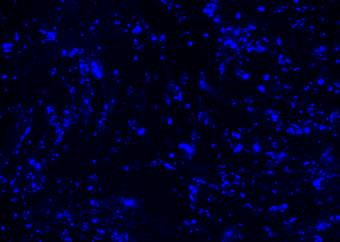

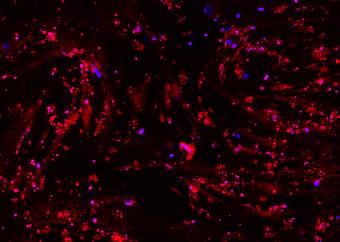


**100 μm**

**CTRL**

**PAK1**

**Supplementary Figure S2. Changes in Mitochondrial Complex Protein and Reactive Oxygen Species (ROS) levels with PAK1 Enrichment. (A)** Validation of Ins R-induced insulin resistance in Ctrl-transduced L6 myotubes via assessment of reduced phosphorylated AKT (p-AKT) relative to total AKT protein (t-AKT). Representative immunoblot and quantitation graph of the ratio of phosphorylated/total AKT protein in L6.GLUT4myc myotubes treated with chronic insulin. Vertical dashed line indicates the splicing of lanes from within the same gel exposure. Data: mean ± SEM. N= 4 independent cell passages per group. **p* < 0.05 (unpaired two-sided Student t test). **(B)** Insulin-resistant (Ins R) ± PAK1-enriched (PAK1-OE) L6.GLUT4myc myotubes were stimulated for 20 min with insulin (100nM) and used in the assays. (n = 4 independent cell passages per group). CTRL, control. Representative immunoblot for mitochondrial Oxphos complex proteins. **(C)** Fluorescent images of superoxide radicals (red). Scale bar=100μm. Blue, nuclear staining.


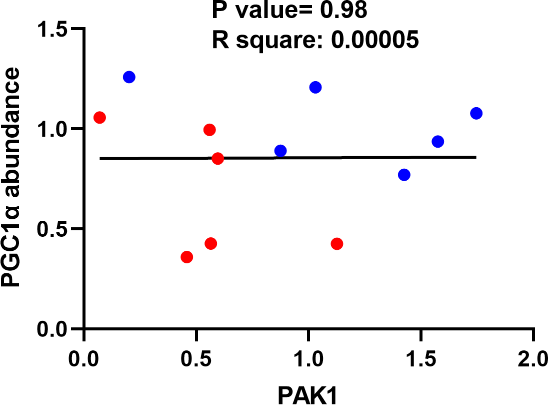


**E)**

**A)**


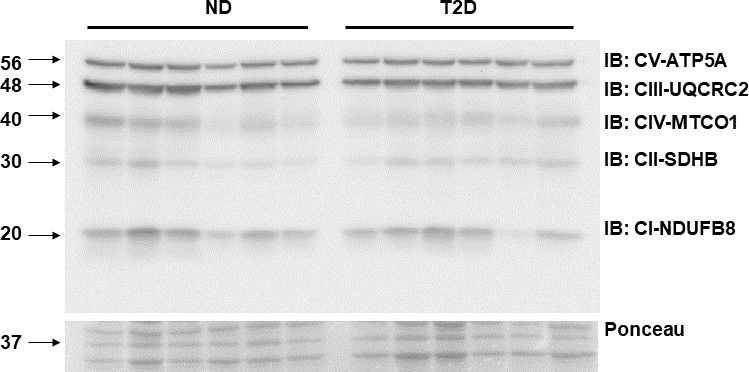


**B)**


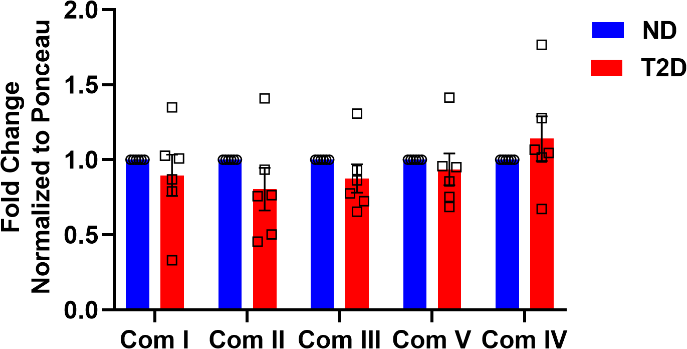


**F)**


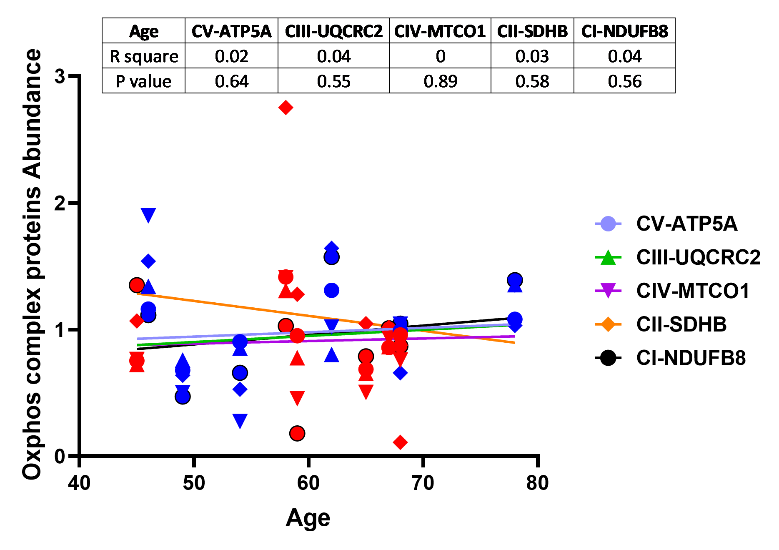


**C)**


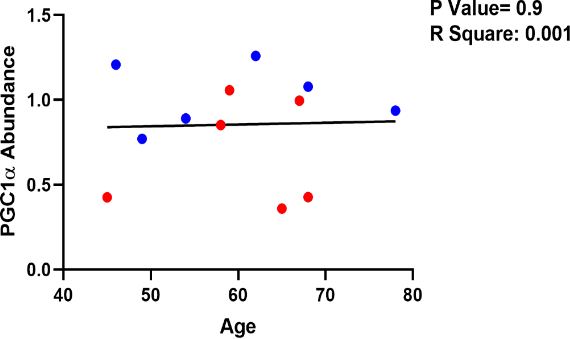

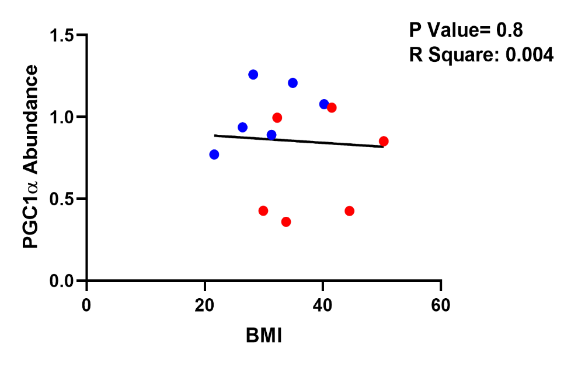


**G)**


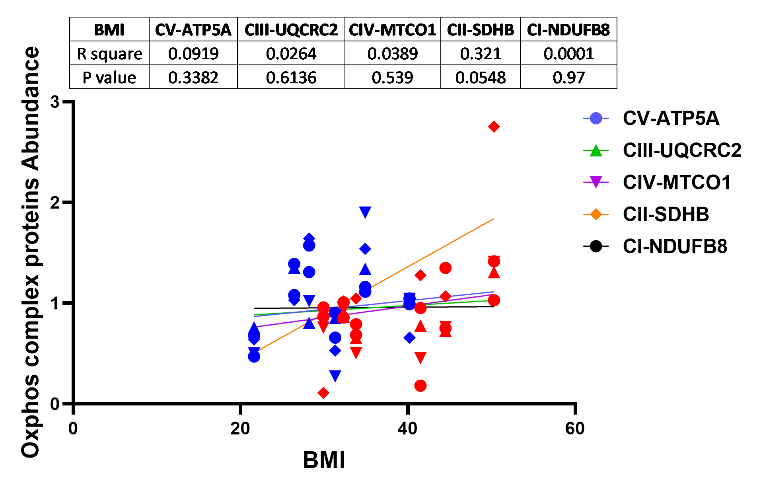


**D)**

**Supplementary Figure S3. Mitochondrial Oxphos complex and PGC1α Abundance in *Human* Skeletal Muscle Samples. (A)** Representative set of mitochondrial oxphos complex proteins from *human* skeletal muscle samples. **(B)** Quantification of each oxphos complex protein. (C-E) Correlation of PGC1α protein abundance in *human* skeletal muscle with Age (C), BMI (D) and PAK1 (E). (F-G) Correlation of Oxphos complex protein abundance in *human* skeletal muscle with Age (F) and BMI (G). (A-G) ND, non-diabetic. ND; n=6, T2D; n=6. (B-G) Linear regression analysis. BMI= Body mass index.


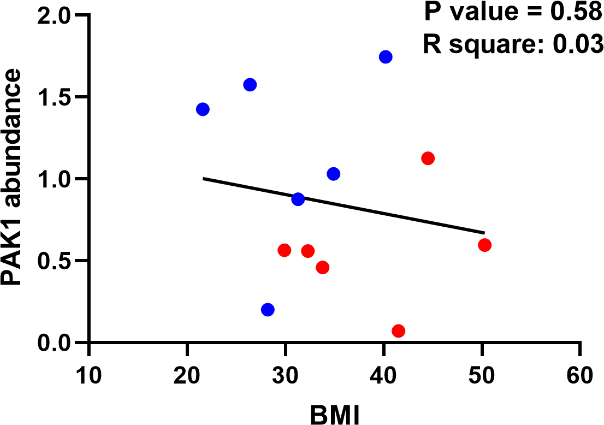


**B)**


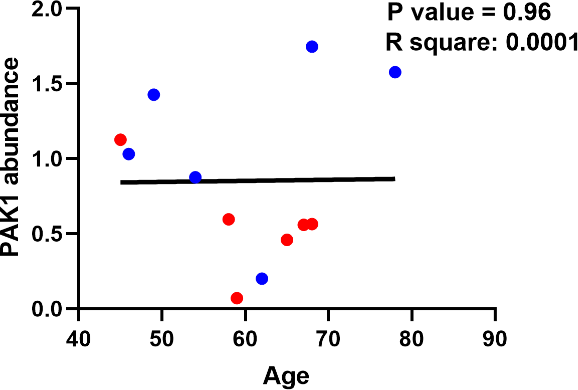


**A)**

**Supplementary Figure S4. Evaluation of PAK1 Abundance Correlation in *Human* Skeletal Muscle Samples.** (A-B) Correlation of PAK1 protein abundance in *human* skeletal muscle with Age(A) and BMI (B). ND, non-diabetic. ND; n=6, T2D; n=6. Linear regression analysis. BMI= Body mass index.

**B)**


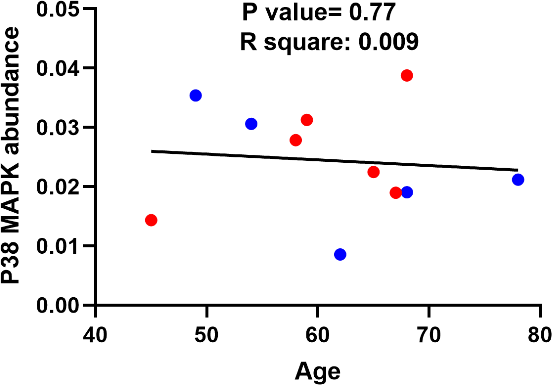

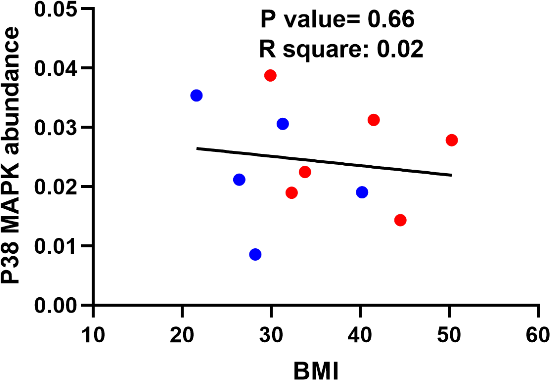
**A)**

**Supplementary Figure S5. Evaluation of P38MAPK Abundance Correlation in *Human* Skeletal Muscle Samples.** (A-B) Correlation of P38MAPK protein abundance *human* in skeletal muscle with Age (A) and BMI (B). ND, non-diabetic. ND; n=5, T2D; n=6. Linear regression analysis. BMI= Body mass index.

**A) B)**


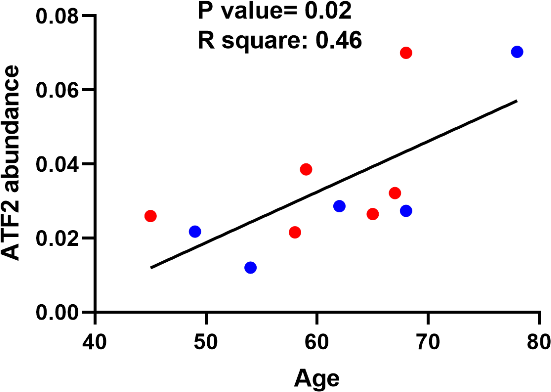

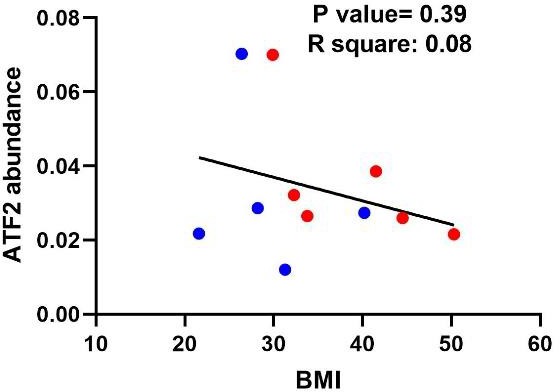


**Supplementary Figure S6. Evaluation of ATF2 Abundance Correlation in *Human* Skeletal Muscle Samples.** (A-B) Correlation of ATF2 protein abundance in *human* skeletal muscle with Age (A) and BMI (B). ND; n=5, T2D; n=6. Linear regression analysis.

BMI= Body mass index.

**A) B)**


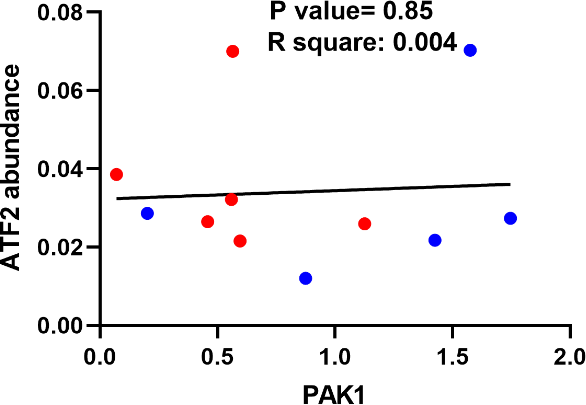

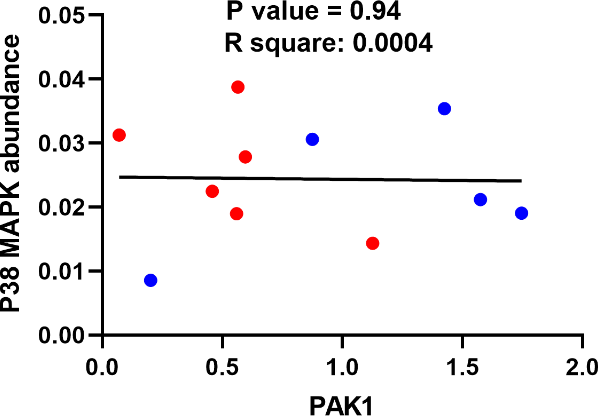


**Supplementary Figure S7. Evaluation of ATF2 Abundance Correlation in *Human* Skeletal Muscle Samples.** (A-B) Correlation of P38MAPK protein abundance in *human* skeletal muscle with PAK1(A) and ATF2 (B) protein abundance. ND, non-diabetic. ND; n=5, T2D; n=6. Linear regression analysis.

**Movies S1 and S2: PAK1 enrichment and insulin stimulation did not alter
nuclear-localized PAK1 levels.**

L6-GLUT4myc myotubes transduced with Ad-CMVGFP (Control) or Ad-CMVmyc-hPAK1 (PAK1) were insulin (100 nM, 20 min) stimulated after 48 hrs of transduction. The z-stack images are compiled to create 3D movies with fixed PAK1 immunostained cells.
